# Physiotherapy students' attitudes toward the use of telehealth in clinical practice: A cross‐sectional survey

**DOI:** 10.1002/hsr2.2067

**Published:** 2024-04-21

**Authors:** Luke Davies, Belinda J. Lawford, Cliffton Chan

**Affiliations:** ^1^ School of Primary and Allied Health Care Monash University Victoria Australia; ^2^ Department of Health Sciences Macquarie University Sydney Australia; ^3^ Centre for Health, Exercise & Sports Medicine The University of Melbourne Melbourne Australia

**Keywords:** curriculum, education, physical therapy, physiotherapy, telemedicine

## Abstract

**Background and Aims:**

Provision of physiotherapy services using telehealth has drastically increased since the COVID‐19 pandemic and continues to be utilized in clinical practice suggesting telehealth in physiotherapy will become common practice. Prior research has explored the attitudes of physiotherapists with many years of in‐person clinical experience toward telehealth. However, little is known about the emerging workforce's attitudes. This study aims to explore physiotherapy students' attitudes toward the use of telehealth in clinical practice.

**Methods:**

A cross‐sectional online survey of physiotherapy students enrolled in the Doctor of Physiotherapy program at Macquarie University between November 2022 and February 2023. Participants rated their level of agreement across 11 statements regarding telehealth use in physiotherapy clinical practice using a 5‐point Likert scale ranging from “*strongly disagree*” to “*strongly agree*.” Participants answered two open‐ended questions regarding when they might use telehealth in clinical practice as a physiotherapist and why they believe physiotherapists might be reluctant to use telehealth in clinical practice.

**Results:**

A total of 118 participants completed the survey (response rate 53%). Overall, most participants believed telehealth would continue being offered post‐Covid‐19 (86%, *n* = 101), participants would use some form of telehealth in clinical practice (82%, *n* 96), believe a blended approach would be beneficial for patients (84%, *n* = 99), and were interested in further training in telehealth (90%, *n* = 107). We identified six broad themes, including accessibility, subsequent consultations, inability to provide manual therapies, limited training/education, perceived ineffectiveness, and digital literacy of the patient.

**Conclusion:**

Overall physiotherapy students believe telehealth will continue being offered in clinical practice, form part of contemporary physiotherapy practice, and are interested in further training to upskill in the delivery of care via telehealth. Given the continued use and students' demand for future training, it may be time to reimagine the inclusion of telehealth education and training in the entry‐level physiotherapy curriculum.

## 1 INTRODUCTION

The provision of physiotherapy services using digital technologies such as telehealth (including telephone, videoconference, and remote monitoring) has drastically increased since the start of the COVID‐19 pandemic and continues to be utilized in clinical practice. Research supports the feasibility of assessment, diagnosis, and management of patients remotely via telehealth.^1^, ^2^ Importantly, compared to in‐person care, physiotherapy services delivered remotely via videoconferencing have shown equivalent and, at times, superior outcomes for conditions such as coronary artery disease, congestive heart failure, surgical populations, as well as musculoskeletal conditions (including post‐knee arthroplasty and knee osteoarthritis).^3^, ^4^, ^5^ Despite the return of in‐person services, evidence shows that physiotherapists and patients will continue using telehealth post‐pandemic.^6^, ^7^, ^8^ This suggests that telehealth use in physiotherapy will become common practice, it is important to understand the attitudes of the emerging physiotherapy workforce toward telehealth in clinical practice.

Entry‐to‐practice physiotherapy training programs routinely focus on developing skills for in‐person assessment and management, often neglecting skill development for working in a digital environment. Telehealth education and training varies substantially across Australian undergraduate physiotherapy programs.^9^ Some universities provide formal telehealth education that is scaffolded across the program, while others provide nothing more than anecdotal discussions during class or nothing at all.^9^, ^10^ A recent report by World Physiotherapy and the International Network of Physiotherapy Regulatory Authorities on digital physical therapy practice highlighted the lack of telehealth education and training provided by entry‐level physiotherapy programs globally.^11^ They advocate that all physiotherapists need to develop digital practice competencies to produce a workforce equipped with the necessary skills and confidence to operate in a digital environment.^11^

Within the physiotherapy profession, the majority of research investigating attitudes toward telehealth in clinical practice has been with experienced clinicians. Broadly, these experiences and attitudes have been positive.^12^ For example, physiotherapists report that telehealth is a great adjunct for care and modernizes how we communicate,^7^ provides greater accessibility to physiotherapy services, promotes self‐management in patients, enables the customization of home exercise programs to better suit patients, and provides flexibility in the delivery of services.^7^, ^13^, ^14^ However, not all physiotherapists share the same perspective, with some viewing telehealth as a temporary stopgap during the COVID‐19 pandemic. Additionally, some physiotherapists prefer assessing and managing patients in‐person, valuing their ability to use a “hands‐on approach” when providing services, and at times feeling estranged from their patients and missing the interpersonal connection that is established in‐person when consulting via telehealth.^7^, ^13^, ^15^ These negative attitudes observed among some physiotherapists are a barrier to the broader implementation of telehealth in clinical practice.

Although existing research has explored the attitudes of physiotherapists with many years of in‐person clinical experience toward telehealth, there appears to be limited research investigating the emerging workforces' attitudes toward telehealth in clinical practice. To our knowledge, no prior research has explored the attitudes of physiotherapy students toward the use of telehealth in clinical practice. Gaining insight into the attitudes and perceptions of students regarding the utilization of telehealth in physiotherapy has the capacity to influence the future direction of telehealth implementation and education. Thus, the aim of this study is to explore the beliefs and attitudes toward telehealth implementation in clinical practice amongst entry‐to‐practice physiotherapy students.

## 2 METHODS

### 2.1 Study design and participants

A cross‐sectional online survey was conducted at Macquarie University between November 2022 and February 2023. Ethics approval was obtained through the Human Ethics Committee at Macquarie University. Physiotherapy students enrolled in any year of the Doctor of Physiotherapy (DPT) program were recruited; a combined cohort size of 221 students. Three recruitment strategies were used. The head of the physiotherapy department was asked to forward an email invitation to all physiotherapy students in the DPT program to participate in the study. Where possible, students were invited to participate in the survey by providing a QR code or hyperlink during a tutorial/lecture at the discretion of the responsible teaching staff. Finally, advertisements were placed on Macquarie University physiotherapy Facebook pages. Only students who were currently enrolled in the 3‐year DPT program at Macquarie University were eligible for this study.

### 2.2 Survey instrument

The checklist for reporting results of internet e‐surveys guided survey development.^16^

Participants completed a purpose‐built online survey developed by the researchers, via Qualtrics, regarding their attitudes on the use of telehealth in physiotherapy clinical practice. Security measures, including relevantID (a functionality that assesses respondent metadata to ascertain the probability that the same respondent is providing repeated responses), bot detection, prevention of multiple submissions, time taken to complete the survey, and the collection of IP addresses were implemented to prevent multiple submissions and identify fraudulent activities. Participants were required to complete an online consent form before accessing the survey. The survey comprised two sections. The first section collected demographic and telehealth experience data such as age, gender, current year enrolled in the DPT program, current clinical placement experience, telehealth training during their program, and the telehealth modalities they would use in clinical practice. The second section related to their opinion on telehealth use in physiotherapy comprising 13 questions that were adapted from previous surveys.^17^, ^18^ There was no operational definition of telehealth provided to students prior or during the survey. Participants were asked to rate their level of agreement across 11 broad questions relating to the use of telehealth in physiotherapy clinical practice and their confidence using a 5‐point Likert scale ranging from 1 being “*strongly disagree*,” “*somewhat disagree*,” “*neither agree or disagree*,” “*somewhat agree*,” and 5 being “*strongly agree*.” Two final open‐ended questions were asked regarding when they might use telehealth in clinical practice as a physiotherapist and why they think physiotherapists may be hesitant to use telehealth in clinical practice.

### 2.3 Data analysis

Incomplete surveys were not included in the analysis of data. Descriptive survey data were downloaded from Qualtrics and imported to the Statistical Package for the Social Sciences (SPSS; Version 28; IBM) software for analysis. Descriptive statistics (frequencies and percentages) were calculated for questions regarding attitudes toward telehealth in clinical practice. To determine the overall attitudes toward telehealth in clinical practice, data were categorized into three groups consisting of “agree” (combined somewhat agree and strongly agree responses), “disagree” (combined somewhat disagree and strongly disagree responses), and “neutral” (neither agree or disagree) for comparative analysis. Responses to open‐ended questions were downloaded from Qualtrics and imported to Microsoft Excel where content themes‐based analysis was performed individually by two authors (L. D. and C. C.) following the steps outlined by Clark, Braun, and Hayfield.^19^ All responses were coded, with similar codes sorted into themes by organizing codes into meaningful groups. There was an independent process of refining codes and collapsing overlapping themes before the two authors met to agree, further refine these, and ensuring all codes were captured in the identified themes. Where the two authors were unable to reach a consensus, the third author was consulted before generating the final themes.

## 3 RESULTS

A total of 118 participants completed the survey (53% response rate). Demographic data for participants are shown in Table 1. More than half the participants were in their second year of the program (54%, *n* = 64), with the majority of participants being male (64%, *n* = 75). Most participants had not received telehealth education and training in the program (89%, *n* = 105). For participants who did receive telehealth education and training in the program, the most common method for delivery was via a tutorial (11%, *n* = 13).

**Table 1:** Participant demographics and characteristics (*n* = 118).

	(*n*, %)
Year of Doctor of Physiotherapy program	
First year	43 (36)
Second year	64 (54)
Third year	11 (10)
Gender	
Male	75 (64)
Female	43 (36)
Age (years)	
18–23	53 (45)
24–29	55 (47)
30–35	8 (7)
35+	2 (2)
Telehealth education/training provided in the program	
Yes	13 (11)
No	105 (89)
Type of telehealth training provided^a^	
A lecture(s)	9 (8)
A workshop(s)	4 (3)
A module(s)	4 (3)
A tutorial(s)	13 (11)
A seminar(s)	4 (3)
Completed clinical placement in the program	
Yes	109 (93)
No	9 (7)
Telehealth practical experience provided on clinical placement	
Yes	33 (28)
No	85 (72)
Type of telehealth you would use in clinical practice as a physiotherapist^a^	
Videoconferencing	120 (101)
Telephone	71 (60)
Remote monitoring (software, apps)	62 (52)

^a^ Does not add to 100% as participants could select multiple types of training that were delivered.

**Figure 1:**
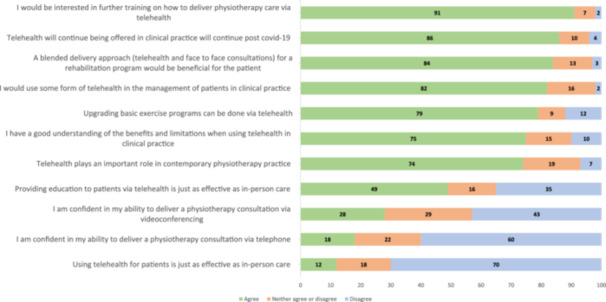
Proportion of agreement to statements regarding beliefs, attitudes, and confidence of telehealth use in clinical practice.

Most participants (86%, *n* = 101) agreed that telehealth will continue being offered in clinical practice beyond the COVID‐19 pandemic and indicated that they would use some form of telehealth in clinical practice to manage patients (Figure 1). Few participants (12%, *n* = 15) agreed that the use of telehealth in clinical practice would be as effective as in‐person care. However, most participants (84%, *n* = 99) agreed that a blended approach to rehabilitation (involving in‐person and telehealth consultations) would be beneficial for patients. Most participants (91%, *n* = 107) indicated interest in attending further training on how to deliver physiotherapy care via telehealth.

### 3.1 Qualitative data

When participants were asked to describe when they might use telehealth in clinical practice as a physiotherapist, two major themes were developed. The themes were accessibility and subsequent consultations.

### 3.2 Accessibility

Participants highlighted the importance of patient access to physiotherapy services, as well as the importance of patients receiving appropriate, targeted, timely, and cost‐effective care. In these instances, telehealth would be useful to increase regularity and adherence to necessary physiotherapy care for people with geographical challenges, other illnesses prohibiting travel or face‐to‐face appointments, and people with varied lifestyle commitments such as looking after children or flexible work schedules. Some quotes that encapsulate this theme include:

“Treating patients in rural or remote settings, treating ‘at risk’ individuals e.g., immunocompromised, treating patients who cannot easily travel/make it to appointments e.g., aged care, full‐time workers” (3rd year, DPT student)

“I think I would use it in circumstances to broaden patient access to physiotherapy. Any patients that find it difficult or inconvenient to physically come to the clinic could really benefit from telehealth. As someone who grew up in a rural area, I can see the potential utility of telehealth for rural patients. Another demographic would be less mobile people, such as those with certain disabilities or housebound for whatever reason.” (2nd year, DPT student)

### 3.3 Subsequent consultations

The use of telehealth for subsequent consultations was strongly expressed. Participants report that telehealth allowed for the provision of education and advice, monitoring of a patient's condition, and supervision and review of home exercise programs, which may ultimately lead to improved patient outcomes and management adherence. Participants also reported that subsequent consultations are better suited to patients who are more confident with performing exercises or demonstrated adequate accuracy in executing instructions provided by their physiotherapist. The following quotes illustrate the nuances of this theme:

“Prescribing exercises through an online software/program, generalised information either video and/or phone. Rehab programming when manual therapy isn't required anymore and you are confident that the patient's technique is correct, e.g. (patient) already has a background in exercise & sports” (2nd year, DPT student)

“In situations involving a quick catch‐up or update on progress is discussed, or the simple progression of an already prescribed program.” (3rd year, DPT student)

“Follow up on patient progress, providing education, answering questions” (1st year, DPT student)

For the question relating to why participants thought physiotherapists are hesitant to use telehealth in clinical practice, four major themes were developed. These were the inability to provide manual therapies, limited training and education, perceived ineffectiveness, as well as digital literacy of the patient.

### 3.4 Inability to provide manual therapies

The inability to perform a complete physical examination or provide manual therapy led to hesitancy in the use of telehealth for physiotherapy consultations. The potential for this to negatively impact or limit the establishment of therapeutic alliances were also an important concern. Examples of these perspectives are reflected in the following quotes:

“I believe there is a fear of patients not feeling like they're receiving treatment. Personally, through clinical placements, I have an appreciation for how much patients enjoy a hybrid style of treatment including some hands on and some exercise. I feel this may be slightly lost if it is strictly telehealth.” (3rd year, DPT student)

“Because there are limitations to going over the web such as measuring with a goniometer and the fact that you can't see properly or feel properly and that you're unable to perform manual therapy if needed. So, you can't really use all your senses over the web and sometimes in physio you kind of need all your senses in a consult which is why some might be hesitant” (2nd year, DPT student)

“Previous physiotherapy beliefs around physiotherapy having to be in person. Miscommunication with patients over video and especially the phone where there is no visuals of the patient‐physio. Decreased patient relationship & therefore rapport. Incorrect rehab movements performed which can recreate and/or exacerbate current injury or even create new injuries.” (2nd year, DPT student)

### 3.5 Limited training and education

The lack of education around telehealth specific skills and use in clinical practice was a strong theme in participant responses. The following quotes illustrate student hesitancy and uncertainty due to a lack of knowledge and practice in telehealth:

“Uncertainty of how to use telehealth and technology in general/not being provided with the knowledge or education on how to utilise telehealth and how it can be beneficial” (2nd year, DPT student)

“Lack of practice/education on delivering effective sessions, may not be able to assess/reassess properly” (1st year, DPT student)

“Lack of educational skills and/or inertia to adapt with the times (old school)” (2nd year, DPT student)

### 3.6 Perceived ineffectiveness

Telehealth was perceived to be ineffective, for physiotherapists themselves as care providers, as well as ineffective for the patients who would receive such care. Physiotherapists were concerned about how their potential patients would perceive telehealth, ultimately affecting patient retention. Participants reported that:

“Traditional physiotherapy seems very ‘hands‐on’ e.g., physical assessments, manual therapies so some clinicians/patients believe that telehealth cannot deliver the same quality of care. I also believe people have an expectation of receiving hand‐on treatment during a physiotherapy session, so could feel cheated/ripped‐off if this wasn't delivered, affecting business.” (3rd year, DPT student)

“As you lose the face‐to‐face interaction. I think there is a powerful placebo when you touch your patients that they begin to feel better. Myofascial release and other massage methods I think are a huge expectation for patients when they come to a private clinic.” (2nd year, DPT student)

“It could compromise the integrity of the assessment and other issues could be missed. Additionally, skills like palpation and providing passive modalities are lost. Lack of understanding could also be a contributing factor.” (2nd year, DPT student)

### 3.7 Digital literacy of the patient

A barrier identified by participants to the wider implementation of telehealth is a patient's digital literacy. Participants report that the use of software, platforms, and camera technology can significantly affect the integrity and quality of physiotherapy care delivered, as demonstrated by the below quotes:

“Unfamiliarity with technology or age of patients and difficulty with technology use.” (3rd year, DPT student)

“It's hard to assess proper exercise technique and complete a whole‐body assessment over Zoom. Very reliant on the tech set up of the patient and how competent they are with tech” (2nd year, DPT student)

## 4 DISCUSSION

The aim of this study was to explore the beliefs and attitudes toward telehealth implementation in clinical practice amongst entry‐to‐practice physiotherapy students. We found that, overall, physiotherapy students have positive perceptions with respect to the role of telehealth and see its continual utilization in contemporary physiotherapy practice. However, some skepticism exists regarding its effectiveness. The low levels of confidence observed by participants in their ability to provide telehealth physiotherapy services may be attributed to a lack of telehealth specific education and training.

Our study shows that a significant proportion of students believe that telehealth will continue being offered in physiotherapy post‐COVID‐19. Moreover, they see value in using a blended approach (in‐person and telehealth) for the delivery of rehabilitation programs, while most indicated they would use some form of telehealth in clinical practice to manage patients. These perceptions are a positive finding given the growing global body of evidence suggesting that physiotherapists and patients themselves intend to continue utilizing telehealth in the future. For example, Australia,^20^ Canada,^6^ Ireland,^7^ Kuwait,^20^ and Switzerland^21^ collected survey data during the pandemic that suggested 20%−89% of physiotherapists intend to continue providing telehealth in clinical practice after the pandemic. In addition, recent studies conducted in the United States^8^ and Australia^12^ revealed that many patients would likely use telehealth, beyond the pandemic, for individual (47%−92%) or group (68%) physiotherapy services. Our study adds to these previous studies' findings suggesting that telehealth utilization will become a more common practice in physiotherapy than it has been in the past.

Our study indicates that many entry‐level physiotherapy students are skeptical of the utility of telehealth use in physiotherapy practice. This is somewhat surprising given that the evidence supports the reliability and validity for performing telehealth assessments^1^, ^2^ and that delivering interventions is safe and as effective as traditional care.^5^, ^22^, ^23^ In addition, less than 30% of students in our study reported confidence in their ability to deliver physiotherapy consultations via the telephone or videoconferencing. Prior research has shown that physiotherapists who receive telehealth training not only view telehealth as an efficient mode of service delivery, but also gain confidence in their ability to adapt their in‐person skills to an online environment.^10^, ^24^ Similar findings are also evident amongst healthcare professionals whose confidence, acceptance, and adoption of telehealth in clinical practice increased with prior exposure and training in telehealth.^25^ Therefore, it could be argued that this is now a critical time to formally incorporate telehealth education and training into entry‐level physiotherapy programs so that students develop the required skills to proficiently work in a digital environment.

Physiotherapy is traditionally a hands‐on profession.^26^ However, we can educate students on how telehealth can be utilized effectively in clinical practice. Our findings demonstrate that many physiotherapy students were interested in upskilling and undertaking further training in the delivery of telehealth in clinical practice. These results echo findings from a previous Australian study involving 343 final year entry‐to‐practice physiotherapy students and new graduates. They found that most participants (89%) reported interest in further training in the delivery of physiotherapy care via videoconference.^18^ This suggests physiotherapy students are interested in the use of telehealth in future clinical practice, recognizing its value, and want to learn how to adapt the skills they are acquiring in‐person to a telehealth setting.

World Physiotherapy, the international voice of over 600,000 physiotherapists worldwide, advocates that digital practice becomes an essential foundational skill and characteristic of future physiotherapists.^11^ It is clear there is a need for educators to implement some form of telehealth education and training into entry‐to‐practice physiotherapy programs to provide students with the foundational skills and knowledge required to practice in a digital environment. A recent study exploring the implementation of telehealth education and training in entry‐to‐practice physiotherapy programs across Australia found that substantial variations exist in the content and volume of telehealth education and training delivered.^27^ For example, some universities scaffold content across the entire program, some include learning outcomes, while others include only an ad‐hoc discussion during a tutorial.^27^ Our findings support this notion showing that most students did not receive telehealth education and training during their program, nor did they have opportunities during clinical placements where telehealth was practically applied. Prior training and experiential learning have been shown to enhance students' understanding, perceptions, and attitudes regarding the utilization of telehealth.^28^ Therefore, future research should focus on the development and implementation of standardized telehealth education modules in entry‐level physiotherapy curriculum, as well as reevaluating recent graduate physiotherapy students' knowledge, skills, and confidence in telehealth utilization.

For a survey‐based cohort study of tertiary students, a response rate above 50% during a period of survey fatigue due to COVID‐19 can be considered reasonable.^29^ However, we need to be cautious with extrapolating our findings and implications as our study represents one postgraduate entry physiotherapy program in metropolitan New South Wales, Australia. Therefore, results may not be generalizable to broader entry‐to‐practice programs, particularly those in rural regions where they may receive more telehealth education and training.

## 5 CONCLUSION

Physiotherapy students believe telehealth will continue to be offered and used in clinical practice post‐COVID‐19. Students also consider telehealth to be beneficial in the management of patients when using a blended approach (face‐to‐face and telehealth consultation) as part of contemporary physiotherapy practice. Furthermore, the majority of students are interested in further training to enhance their competency in delivering physiotherapy services via telehealth. Given the continued use of telehealth in clinical practice and there is clear demand that students want training in the delivery of telehealth services, it may be time to reevaluate the physiotherapy curriculum.

### 5.1 Implications of physiotherapy practice

This study suggests that entry‐level physiotherapy students recognize the advantages that telehealth can provide in clinical practice, intend to use telehealth to manage patients in their future careers, and are interested in further training in telehealth delivered physiotherapy. It is important for entry‐to‐practice programs to implement such training to ensure that the future physiotherapy workforce has the necessary skills to competently work in a telehealth environment.

## AUTHOR CONTRIBUTIONS

**Luke Davies**: Conceptualization; data curation; formal analysis; methodology; writing—original draft; writing—review and editing. **Belinda J. Lawford**: Writing—original draft; writing—review and editing. **Cliffton Chan**: Formal analysis; writing—original draft; writing—review and editing. All authors have read and approved the final version of the manuscript

## CONFLICT OF INTEREST STATEMENT

L. D. and C. C. are employed within the Department of Health Sciences, Faculty of Medicine, Health and Human Sciences at Macquarie University. The remaining author declares no conflict of interest.

## ETHICS STATEMENT

Ethics approval was obtained through the Human Ethics Committee at Macquarie University (12306). Participants were invited to participate and presented with a plain language statement detailing their confidentiality and freedom of participation before consenting to participate in the survey.

## TRANSPARENCY STATEMENT

The lead author Luke Davies affirms that this manuscript is an honest, accurate, and transparent account of the study being reported; that no important aspects of the study have been omitted; and that any discrepancies from the study as planned (and, if relevant, registered) have been explained.

## Data Availability

The data that support the findings of this study are available on request from the corresponding author. The data are not publicly available due to privacy or ethical restrictions.
